# A Vision-Based System for Intelligent Monitoring: Human Behaviour Analysis and Privacy by Context

**DOI:** 10.3390/s140508895

**Published:** 2014-05-20

**Authors:** Alexandros Andre Chaaraoui, José Ramón Padilla-López, Francisco Javier Ferrández-Pastor, Mario Nieto-Hidalgo, Francisco Flórez-Revuelta

**Affiliations:** 1 Department of Computer Technology, University of Alicante, P.O. Box 99, Alicante E-03080, Spain; E-Mails: jpadilla@dtic.ua.es (J.R.P.-L.); fjferran@dtic.ua.es (F.J.F.-P.); mnieto@dtic.ua.es (M.N.-H.); 2 Faculty of Science, Engineering and Computing, Kingston University, Penrhyn Road, Kingston upon Thames KT1 2EE, UK; E-Mail: F.Florez@kingston.ac.uk

**Keywords:** intelligent monitoring, vision system, ambient-assisted living, human behaviour analysis, human action recognition, multi-view recognition, telecare monitoring, privacy preservation, privacy by context

## Abstract

Due to progress and demographic change, society is facing a crucial challenge related to increased life expectancy and a higher number of people in situations of dependency. As a consequence, there exists a significant demand for support systems for personal autonomy. This article outlines the vision@home project, whose goal is to extend independent living at home for elderly and impaired people, providing care and safety services by means of vision-based monitoring. Different kinds of ambient-assisted living services are supported, from the detection of home accidents, to telecare services. In this contribution, the specification of the system is presented, and novel contributions are made regarding human behaviour analysis and privacy protection. By means of a multi-view setup of cameras, people's behaviour is recognised based on human action recognition. For this purpose, a weighted feature fusion scheme is proposed to learn from multiple views. In order to protect the right to privacy of the inhabitants when a remote connection occurs, a privacy-by-context method is proposed. The experimental results of the behaviour recognition method show an outstanding performance, as well as support for multi-view scenarios and real-time execution, which are required in order to provide the proposed services.

## 1. Introduction

Video cameras are commonly used in video surveillance systems in order to guarantee security in outdoor environments and public places. However, due to the nuisance of being continuously monitored and the concern of losing our right to privacy, such systems have only sparingly reached private places, such as our homes. Nonetheless, if vision-based information related to human activity at home were provided, a vast amount of services regarding entertainment, home automation, security and safety, among others, could be deployed, since cameras provide rich sensory information. Specifically, in ambient-assisted living (AAL), advanced support and care applications can be developed relying on vision-based technology [1,2]. In AAL, ambient intelligence is applied to the promotion and extension of independent life at home for elderly or impaired people. In this sense, AAL can give diverse types of support to ensure people's health and safety, and to increase their autonomy and well-being, by means of providing services from the automatic supervision of medication to intelligent monitoring. These services are nowadays in great demand, due to the rapidly ageing populations. The European Statistical Office projects that by 2060, the ratio between working and retired people will have passed from four-to-one to two-to-one in the EU. In addition, EU Member States spend nowadays approximately a quarter of their GDP on social protection [3]. Such a demographic and economic context raises the concern of whether these high standards can be maintained. For this reason, allowing people to stay active and independent as they grow older is key to tackle the challenge of demographic ageing.

In this regard, the vision@home project (a related conference publication has been presented in the last International Work-conference on Ambient Assisted Living (IWAAL 2013)) [4] aims to increase the personal autonomy of elderly and impaired people by means of supporting AAL services, such as, for instance, the detection of home accidents, long-term behaviour analysis, telecare and telerehabilitation. In this intelligent monitoring system, mainly visual information is considered to perform human behaviour analysis. Multiple-camera scenarios and different visual sensors (both RGB and RGB-D) are supported for the recognition of human actions, like walking, sitting or falling, enabling not only the detection of short-term events, but also long-term behaviour monitoring. If the detected event corresponds to a situation of risk, a caregiver can be informed and risk assessment can be carried out. For this purpose, privacy protection is taken into account, making it possible to share visual information, while preserving the right to privacy of the inhabitants.

In this paper, the vision@home project is presented regarding the specification, design, development and experimentation stages. The proposed architecture of the intelligent monitoring system considers multimodal sensory information, *i.e.*, visual sensors, as well as environmental sensors. Single- and multi-view human behaviour analysis is proposed as a knowledge base in order to support different kinds of AAL services for the inhabitants, from fall detection to long-term human behaviour analysis. Particularly, a human action recognition method based on silhouette motion is presented, proposing novel techniques for the fusion of multiple views and data acquisition. Telecare services are also supported by means of an external visual connection to the cameras. Taking advantage of the performed behaviour recognition, a privacy-by-context technique is proposed to modify the original image, concealing different details to preserve the right to privacy of the persons using a level-based visualisation scheme.

The remainder of this paper is organised as follows: Section 2 summarises existing work related to monitoring systems, human action recognition and privacy protection techniques. In Section 3, the requirements and proposed design of the intelligent monitoring system are presented. Section 4 details the employed human action recognition method, proposing new techniques for learning and recognition with multiple views. Section 5 specifies the privacy-by-context method that is presented to alter the image that can be observed by other persons, like caregivers or relatives. In Section 6, the experimental results of the human action recognition proposal on which this system is based are detailed. Furthermore, an alternative data acquisition method is proposed. Finally, Sections 7 and 8 provide a discussion and conclusions.

## 2. Related Work

In this section, the most recent and relevant related work is summarised regarding the different proposals made in the vision@home project. Existing video-based monitoring systems are detailed considering both video surveillance and home monitoring systems and focusing on their consideration of privacy. Then, human action recognition methods are analysed, taking into account specifically multi-view methods. Finally, the context of relevant privacy methods is given.

### 2.1. Video-Based Monitoring Systems

The most popular application of visual monitoring is certainly video surveillance. A huge amount of cameras have been installed in the last few decades, and the need for intelligent surveillance systems has appeared, since constant manual monitoring is not feasible. In this sense, great advances have been made so far. For instance, people and object tracking for outdoor environments are supported by the surveillance systems, W^4^ [5] and the IBM Smart Surveillance System (S3) [6]. The former can detect and track multiple people under occlusions and monitor their activities, such as, for instance, leaving a bag at the airport, exchanging a bag among two persons or removing an object (theft). A silhouette analysis of the person is performed in order to detect body parts (head, hands, feet and torso) and to manage people and object interactions. However, the latter enables the use of several event analysis technologies that are indexed into a common repository. S3 supports number plate recognition, object detection, tracking and classification and face detection and recognition. Moreover, this system supports surveillance data managing, event-based retrieval, real-time event alerts and the extraction of long-term activity patterns.

Conversely, in relation to the previously described systems, PriSurv [7] does not intend to recognise people's activities, but to preserve their privacy. It is a surveillance system for small communities, where members sometimes act as viewers and, other times, as monitored subjects. Therefore, privacy policies are determined according to the closeness between monitored subjects and viewers. Different visual abstract representations are applied accordingly. People identification is carried out through RFID tags and image processing in order to retrieve their privacy policy. Similarly, the Respectful Cameras [8] system detects visual markers in real time to address privacy concerns. Because of the usage of markers, this system supports dynamic backgrounds and camera movements. People indicate their privacy preferences by means of coloured markers, such as hats or vests. Once markers have been detected, this system obscures the detected faces with solid ellipsoidal overlays. A different approach is taken in [9], where a smart camera that provides security and preserves privacy based on trusted computing is presented. TrustCAM digitally signs video streams in order to detect the manipulation of the recorded videos. Furthermore, sensitive data, such as faces or the number plates encrypted, and an abstracted version of that data, like silhouette representation, can also be generated and encrypted.

Regarding specifically intelligent monitoring systems for AAL purposes, the main differences that can be found with respect to traditional video surveillance systems rely on the following two facts: (1) a different set of behaviours have to be recognised, which can lead to specific constraints (e.g., fall detection instead of vandalism); and (2) homes or nursing centres are made up of small indoor spaces, where different restrictions apply to camera views. For example, in [10], a vision-based health monitoring system is proposed. This system learns the activity patterns of the elderly and detects anomalies or deviations that may suppose a change of health status. Activities are recognised by visiting predefined activity zones in the environment. When a subject stays in an activity zone for more than one second, the event is logged. Pattern analysis is performed later in order to detect deviations from the learned activity pattern. Concerning the detection of home accidents, Altcare [11] is a vision-based monitoring system for the detection of emergencies at home. This system is especially focused on fall detection. When a fall is detected, the system attempts to confirm it with the involved person before triggering an alarm. Afterwards, if there is a positive response or no response at all, it communicates the emergency to the person in charge. A patch of the video showing the emergency is transmitted, where the person is replaced by the silhouette, so as to preserve privacy. Moreover, if so desired by the monitored person, an administrator may connect at any time in order to check the state of the person.

### 2.2. Human Action Recognition

Human action recognition deals with the essential task of recognising short temporal intervals of human behaviours, like walking, running or sitting down, which are part of a great number of activities of daily living (ADL). While significant advances have been made in monocular computer vision systems, huge difficulties remain in achieving the desired robustness and generality of vision-based human action recognition [12]. Current efforts rely especially on reaching real-time performance [13] and taking advantage of multiple views in order to improve the recognition [14]. For this purpose, also alternatives to RGB images are being explored, working especially with depth sensors (RGB-D cameras, such as the Microsoft Kinect), which allow one to obtain 3D representations and markerless body pose estimations [15].

Regarding multi-view human action recognition and how the information from multiple views is combined and fused, different levels at which the fusion process happens can be found. The most straightforward option, known as decision-level fusion, consists in using single-view action recognition methods individually for each view. Once all the recognised classes are returned, the result is reduced to a single recognition value. Commonly, the best view is chosen based on some input/output criteria as the view with the biggest region of interest [16], the highest probability of feature matching [17] or the highest accuracy obtained during training [18]. In [19], Naiel *et al.* used a majority voting technique, where the camera with a minimum distance is chosen when no majority is reached. In this work, the well-known motion history and energy images (MHI, MEI) from [20] are reduced with a two-dimensional principal component analysis (PCA) and classified based on a k-nearest neighbour classifier. Zhu *et al.* go further in [21], where the decision is based on the accumulation of each view's weighted prediction histogram. The prediction histograms are obtained by mapping the local segment features of binary silhouettes using a random forest classifier. Similarly, in [22], a consensus-based distributed algorithm is proposed that relies on matrix completion. In comparison with the former, these approaches have the advantage that all the available viewing angles contribute to the final result. This can potentially improve the recognition of actions whose best view may change during its performance (e.g., due to occlusions or the type of action itself).

In order to exploit the inherent relation between multiple views of the same field of view, the action recognition method needs to learn from multi-view data. Model fusion can either happen implicitly, by feeding the learning algorithm with images from multiple views, but ignoring their viewing angle, or explicitly, by changing the learning scheme, so as to support multiple views. In the case of [23], both MHI and the frame descriptor from Tran and Sorokin [24] are tested. Using dimensionality reduction techniques, the features are matched with a probabilistic classifier, and the final multi-view frame recognition is performed by means of a Bayesian network. Action sequence recognition is then handled using a discriminative hidden Markov model (HMM).

A simpler way of considering multiple views is to perform feature fusion. Combining several single-view features in order to obtain a multi-view descriptor enables one to use single-view human action recognition methods with the least number of changes. Nonetheless, in order to achieve the desired performance, both the feature type and the classification method need to be suitable, since feature fusion techniques may prohibitively increase the dimensionality of the feature descriptor and make difficult the discrimination. Different types of feature fusions can be found. In [16], an aggregation function (average value) is tested, which, in fact, outperforms other approaches based on decision-level fusion. Conversely, in [25], single-view features are successfully joined using a concatenation of vectors and, therefore, preserving all the characteristic data. More sophisticated techniques can also be found, as in [26], where canonical correlation analysis is employed.

The lowest level at which information fusion may be carried out is data fusion. This means that, prior to any feature extraction, data from multiple views is combined together in order to generate a 3D representation, as in [27]. This type of fusion allows one to avoid the increased information loss coming from multiple feature extractions from raw data.

Finally, these levels of information fusion can be combined. For instance, recently, in [28], a learning scheme has been presented that fuses labels at three different levels: blocks of features, camera-specific labels and frame-specific labels. Good results are achieved on the IXMAS dataset [29].

### 2.3. Visual Privacy Protection

A camera provides rich sensory information in the form of visual data. This data conveys a lot of visual clues about individuals appearing in the scene, which can be automatically extracted by means of computer vision and machine learning techniques. Furthermore, most of this data may be considered as private by individuals. Therefore, privacy protection methods are needed in order to preserve individual's right to privacy. However, privacy is a very subjective issue, because it depends on a variety of factors: closeness between the monitored person and the viewer, information that is considered to be private (e.g., identity, appearance, location, activity, *etc.*), one's own sense of privacy, and many others. Nevertheless, we have to distinguish between the identity of the person and the related sensitive information. If this information is known, but there does not exist any association between them, the privacy is preserved.

Different methods for protecting the privacy of individuals in videos and images can be found in the literature [7,30]. There are two main approaches, depending on when privacy protection is performed: (1) before image acquisition (intervention); or (2) after image acquisition (image redaction). The first one consists of preventing others from taking photos by physically intervening in the camera's optical lens. Software methods for preventing a camera from taking photos under some circumstances could also be included here. The second approach consists in modifying or removing sensitive areas (e.g., faces, number plates, *etc.*) from the original image before it is going to be stored, distributed, shared or visualised. Since the original image cannot be recovered in some cases, often, image redaction methods are used along with data hiding algorithms in order to conceal the original image inside the modified one for later retrieval, if needed. Image redaction methods can be classified in several groups concerning the way the original image is modified: (i) image filters that apply visual effects, like blurring or pixelating; (ii) image encryption and scrambling that ciphers the image in order to make it unintelligible; (iii) face de-identification based on the k-same family of algorithms that modify facial features; (iv) object and people removal using image inpainting; and (v) object and people replacement by visual abstractions.

Finally, image modification methods have to tackle the image usefulness issue in fields like video surveillance or telecare monitoring, *i.e.*, if an image is modified, information needed for image understanding may be also removed. Therefore, a balance between privacy and image usefulness is required.

## 3. Intelligent Monitoring System

An intelligent environment needs to be aware of the events that are happening in order to assist proactively. This assistance can be of any kind, from closing a running tap to firing an alarm or calling the telecare call centre. Not only short-term events are interesting, but also the long-term behaviour of the persons. Action recognition allows one to collect information about the daily living of people, which can make it possible to learn routines and recognise abnormal situations, thus allowing the early risk detection of health issues. This kind of knowledge is valuable in order to offer AAL services. Furthermore, by means of employing vision techniques, telecare services, like telecare monitoring or telerehabilitation, can be offered.

Nowadays, a lot of application scenarios have multiple cameras available. Due to the reduction of costs and the increase of popularity of outdoor and indoor cameras, there are commonly several cameras installed covering the same field of view. Especially in human action recognition, one camera can be insufficient, due to partial occlusions (objects like furniture could be in the way, but also other persons) and ambiguous or unfavourable viewing angles. Several video streams can be analysed, and multi-view representations can be modelled to improve action recognition. However, there are still some important difficulties to overcome, as dealing with multi-view data leads to high computational cost and burdensome systems [14]. The main reasons for this situation are: (1) the additional increase of difficulty in learning from multiple views, because the combination of multi-view data results in a greater data variance and complex learning models; and (2) the involved decrease of recognition speed, since at least two views need to be processed and analysed (or chosen from).

Below, the objectives of the intelligent monitoring system of the vision@home project are specified as the AAL services the system should be able to support. Based on these, the requirements of the system are briefly summarised, and a suitable architectural design for this monitoring system is proposed.

### 3.1. AAL Services and Requirements

As has been mentioned beforehand, the main goal of the vision@home project is to support personal autonomy in order to extend the independent living at home of elderly and impaired people. For this purpose, two categories of AAL services are considered to provide care and safety: (1) intelligent monitoring services; and (2) video-based telecare services. Among the first category, services, such as home accident and risk detection (e.g., falls and disorientation), recognition of ADL (related to physical and mental health status) or long-term behaviour analysis and abnormal behaviour detection, can be found. Regarding video-based telecare services, mainly risk assessment and safety confirmation, as well as telerehabilitation and videoconferences are taken into consideration.

In order to accomplish these kinds of objectives, different requirements have to be considered. Since the proposed AAL services rely on visual information and its analysis, the system needs to perform video-based monitoring in real time to recognise motion-based human actions, like walking, running, falling, bending, standing up, sitting down, *etc.*, as well as more complex activities of daily living. The recognised activities have to be saved in order to support long-term analysis of the human behaviour. The system should be able to learn from multiple views, if these are available. Furthermore, since training and testing setups may change, it has to be taken into account that a view may not be available during the recognition. In addition, in homes and environments, in general, it is very difficult to install specific camera setups. Therefore, relaxed multi-view requirements are needed. Camera calibration, specific viewing angles and a constant number of views cannot be guaranteed.

Moreover, the system must provide a visual connection to an authorised remote observer (e.g., healthcare professionals, telecare call centre and relatives) as part of the telecare services. In this case, the privacy of the persons appearing in the scene must be protected in the images. Furthermore, because of the subjectivity involved in privacy, a privacy protection method is needed that must be able to adapt to the individual sense of privacy and, at the same time, include the appropriate information in order to ensure that the AAL service can be provided. As a result, the privacy method must protect identity, appearance, location and ongoing activity.

### 3.2. Architectural Design

In order to support the detailed AAL services and considering the resulting requirements, an architectural design for the intelligent monitoring system is presented in the following.

The proposed system relies on a multi-view setup of cameras installed in people's homes in order to apply human behaviour analysis at two levels: single and multi-view. For each room, these cameras cover a shared field of view. Based on motion detection, image processing tasks, such as background segmentation, will be applied to extract characteristic information from the video streams. Human behaviour analysis is then performed considering both single- and multi-view recognition with the goal of providing a semantic meaning to the observed human activity.

At higher processing levels, the visual information is fused with the information provided from environmental sensors (motion detectors, pressure sensors, proximity sensors at doors, windows and cupboards, thermometer, gas, fire and flooding sensors, *etc.*). This provides essential data in order to recognise more complex activities, such as those that require interactions with objects in the environment. Furthermore, they can provide verification data related to human activity in order to confirm visually recognised behaviour. A reasoning system then decides if the detected activity corresponds to any of the monitored events, such as falls and specific actions, and acts accordingly, for instance, firing an alarm or enabling certain actuators.

Furthermore, the proposed design also considers ethical issues in order to preserve the privacy of the inhabitants by means of image post-processing and permission management, among other techniques. In this way, a caregiver can be informed of the event that occurred if it is necessary. This may be a professional tele-assistance service that is directly connected to the home or an informal caregiver (e.g., a relative) that can be informed with a text message. By means of the privacy layer, the appropriate textual or visual information can be shared depending on the permissions of the observer and the risk involved in the detected event. In this regard, sensitive information or even the identity of the person will be modified or removed in order to preserve the privacy before the information is shared.

**Figure 1.**
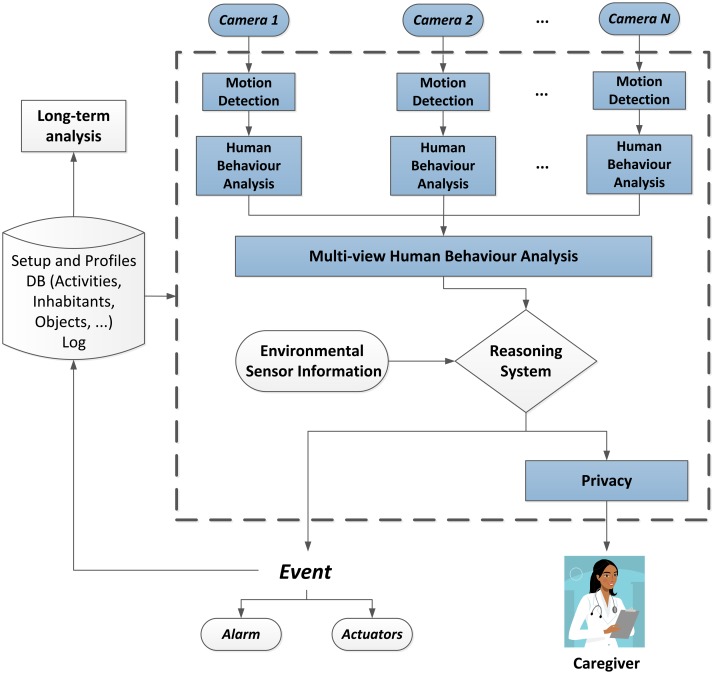
Architecture of the intelligent monitoring system to promote independent living at home and to support AAL services. The parts that are further specified in this contribution are shaded in blue. (DB stands for database.)

The results of the monitoring are logged 24 hours a day, making it therefore possible to perform a long-term analysis of the human behaviour based on this data. This leads to support, as well as abnormal behaviour detection. Figure 1 shows a diagram of the complete architecture of the system, where the parts that have been developed so far are highlighted. These will be further detailed in the following sections.

## 4. Human Action Recognition

Our proposal for human behaviour analysis addresses particularly human action recognition. The method that will be laid out in this section is based on previous work, where a classification method for human actions based on a bag of key poses model [31,32] and a silhouette-based feature that relies on a radial scheme [33] have been presented. In order to improve the learning from multiple views and overcome shortcomings present in previous proposals, next, enhancements are presented for both pose representation and recognition stages.

### 4.1. Pose Representation Stage

In the following, a summarised description of the employed single-view pose representation is detailed in order to present a novel method for the fusion of multiple views afterwards.

#### 4.1.1. Single-View Pose Representation

As has been mentioned before, the proposed human action recognition method relies on a silhouette-based pose representation. The choice of this type of input data is motivated by its rich posture-based shape information, as well as its reduced computational cost of processing. Once the silhouette of the person is obtained from a video frame, this global shape representation can be simplified in a single array of points representing the boundary of the human silhouette. In the next step, a radial scheme is applied to divide the shape of the body spatially and to process the contour points in a radial fashion. In contrast to other similar contour-based approaches, this important step achieves the performance of spatial alignment, since body parts fall in the same radial bins for similar poses. This is not the case for a point-wise extraction, where slight differences can significantly change the order. Based on this division in *B* radial bins, we propose to extract a single representative value for all the contour points of each bin. These summary values are based on the statistical range of distances between each contour point and the centroid. This simplifies the two-dimensional point data to a one-dimensional real value and achieves location invariance. Finally, scale normalisation is applied, and the final feature vector is generated using the concatenation of summary values. In fact, this feature, used along with its evolution over time, has proven to be proficient for silhouette-based action recognition and feature selection (we refer to [33] for greater detail).

**Figure 2.**
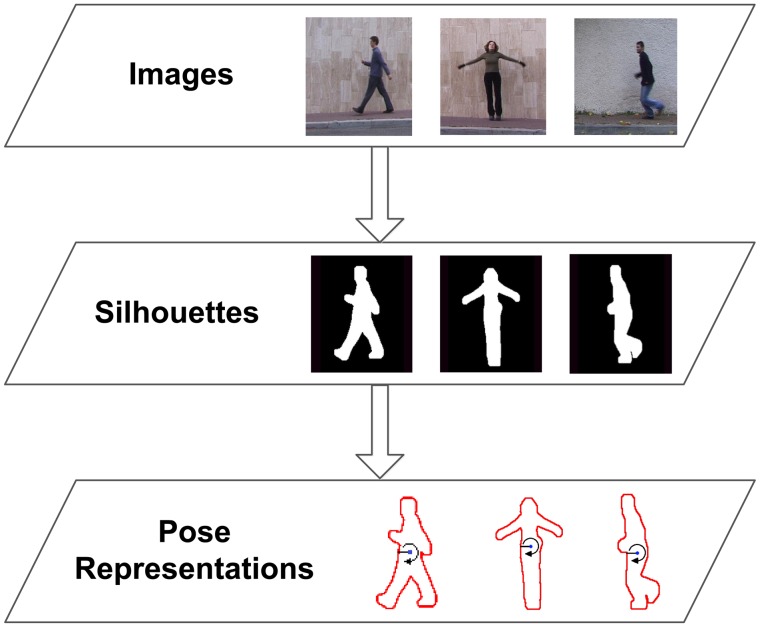
Outline of the pose representation process. Based on the recorded video frames, foreground segmentations are obtained. Relying on the shape of the human silhouettes, a low-dimensional and radial feature is extracted.

Since the method only relies on the boundary points of the human silhouette in order to obtain a contour-based feature, the actual RGB image is not always needed. As will be seen in Section 6, we use human silhouettes acquired through background subtraction or depth-based segmentation. However, the boundary of the human shape could also be obtained from infra-red, thermal and laser-based cameras, which enable other privacy preserving techniques. Figure 2 shows a diagram of the processing stages that have to be performed for feature extraction.

#### 4.1.2. Multi-View Pose Representation

Multi-view human action recognition can provide several benefits over a single-view approach. Having multiple images covering the same FOV allows one to increase the robustness to occlusions, ambiguous viewing angles, noise or sensor failure, just to name a few. Nonetheless, only a small number of methods handle multi-view scenarios successfully [14]. The main problems encountered are the increased difficulty of the classification of multi-view data and the involved performance issues.

In previous work, both feature fusion and model fusion have been considered. Whereas accurate recognition results have been achieved with the latter option, it presented a decreasing performance when the number of views rose. Furthermore, at the testing stage, the views were individually evaluated. Therefore, the evaluation time increased with the number of available views in a linear fashion. On the other hand, the feature fusion approach does not allow one to obtain the best performing view, since all the features are equally considered.

**Figure 3.**
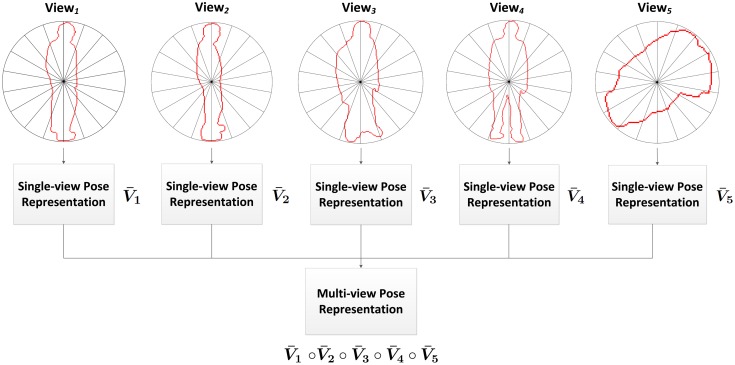
Overview of the generation process of the multi-view pose representation. This example shows a specific pose taken from the walk action class from the IXMAS dataset.

In this work, an intelligent feature fusion approach is proposed in order to combine the advantages of both approaches. The aforementioned feature is obtained for each of the available views, and the multi-view pose representation is generated afterwards by means of combining these features into a single feature descriptor. Specifically, a concatenation of single-view features has been chosen in addition to a weighting scheme (see Figure 3).

##### Weighted Feature Fusion Scheme

Some viewing angles may be more or less useful than others depending on the captured images. Intuitively, the front camera should provide information with a greater characteristic value than the one that is recording the rear, since actions are normally performed towards the front. On the other hand, they may be equally useful if actions are performed sidewise. In order to confirm this idea, single-view recognition results have been studied. It has been observed that, in general, lateral views perform similarly when several action classes are considered, and only the top view returned steadily worse results (see Table 1). This behaviour can be explained regarding the different types of actions. Each one of them may be recognised more easily from different views depending on the involved motion and on the position and orientation of the subject. Therefore, two conclusions can be stated. First, the usefulness of each view must be obtained automatically in the learning process, since it depends on the particular camera setup. Second, the specific weights to be assigned to each view depend on the action to recognise, since different action classes may be recognised best from different viewing angles.

In this sense, a weighted feature fusion scheme is proposed, which uses specific weights for each view and action class and takes them into account in the comparison of features. Thus, intelligent information fusion can be applied using *a priori* knowledge about the input data.

Let us suppose that there are *M* camera views available, and the method is learning *A* action classes. The camera weights are obtained as follows:

(1) $$\begin{array}{cccc} w_{m,a}=\text{Test}{\left(m\right)}_{a}, & \forall m\in [1\ldots M] & \wedge & \forall a\in [1\ldots A] \end{array}$$

where Test(*m*) evaluates the recognition of the *A* action classes in a single-view test using only view *m* and returns an array of the recognition results of each action class. Therefore, the per-class success rates of each camera are used as weights in order to determine how useful a camera is at recognising each of the action classes. Finally, these weights are normalised to a unit-sum.

Last, but not least, camera setups are subject to change. This method does not rely on camera calibration, and small viewpoint variations are inherently supported by the contour-based feature. Each camera view is explicitly considered in the feature fusion, by preserving the origin of the individual feature part. In case that a fewer number of cameras were available in the test scenario, only the matching camera views would be compared. Thus, recognition can be performed if at least one of the camera views used during training is available.

### 4.2. Learning Stage

Since the learning stage has remained the same as the one that has successfully been employed before, we refer to [32] for greater detail and include a short description below.

In order to retain the most representative data, key poses are obtained. Key poses allow one to recognise a human action based on a few indicative poses and make it possible to omit non-relevant or redundant pose information. In this sense, for each action class, the available multi-view training sequences are taken, and their silhouettes are processed to obtain the corresponding multi-view pose representations. These features are reduced to *K* representative instances using *K*-means clustering. The returned cluster centres are used to create the key poses, *kp*_1_…*kp_K_*, involved in each action class. These multi-view key poses are then joined together in the same bag of key poses model, which serves as a dictionary of the most relevant poses across action classes.

**Figure 4.**
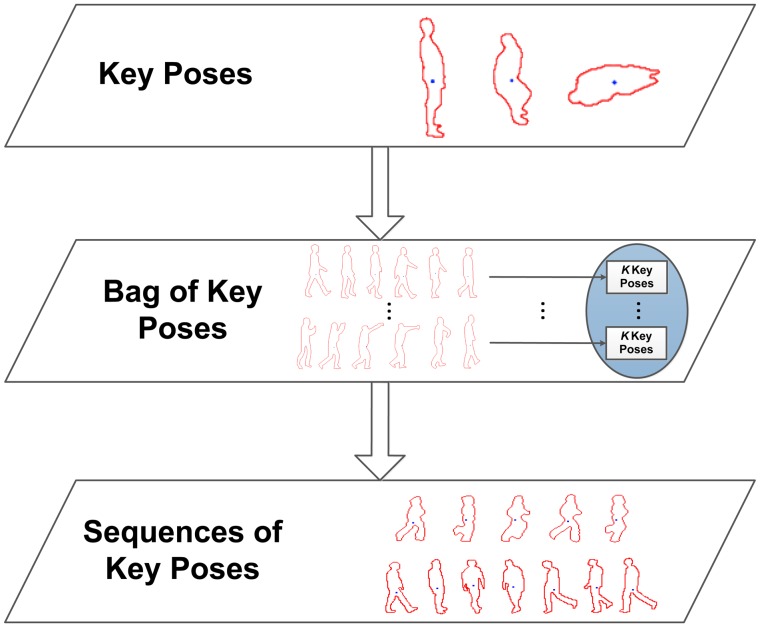
Outline of the learning stage. Using the pose representations, key poses are obtained for each action. In this way, a bag of key poses model is learned. The temporal relation between key poses is modelled using sequences of key poses.

So far, no temporal aspects have been taken into account. Therefore, at this point, the goal is to learn sequences of key poses in order to model their temporal evolution along action performances. For this purpose, for each element of a sequence of multi-view pose representations, the corresponding nearest neighbour multi-view key pose out of the bag of key poses model is sought. The successive matched multi-view key poses build up sequences of key poses, *i.e.*, *Seq* = {*kp*_1_, *kp*_2_, …, *kp_t_*}. In this way, the long-term temporal relationship between key poses is modelled, and furthermore, particular instance- and actor-related noise and outlier values are filtered. Figure 4 shows a diagram of the detailed processing stages.

### 4.3. Recognition Stage

For the recognition stage, we propose to perform a recognition of human actions taking into account the previously learned viewpoint relevance. For the purpose of classifying sequences of key poses, sequence matching is used in order to find the closest sequence using temporal alignment.

Given a new multi-view video sequence to recognise, initially, the same procedure as with the training instances is performed:

1. The video frames from each view are processed to their single-view pose representation.
2. The multi-view pose representations are obtained as described in Section 4.1.
3. The equivalent sequence of key poses ${\text{Seq}}^{\prime }=\{kp_{1}^{\prime },kp_{2}^{\prime },\ldots ,kp_{u}^{\prime }\}$ is built by replacing each multi-view pose representation with its nearest neighbour multi-view key pose.

Dynamic time warping (DTW) has been chosen for the comparison of sequences. DTW finds the temporal alignment of two sequences that presents the minimal distance and is therefore able to align patterns that are shifted due to non-uniform speeds. This is essential for human action recognition, since the pace at which an action is performed depends on the age and condition of the person.

The required DTW distance, *d_DTW_*(*Seq*, *Seq*′), is obtained as follows:

(2) $$\text{dtw}(i,j)=\min\;\left\{\begin{array}{l} \text{dtw}(i-1,j),\\ \text{dtw}(i,j-1),\\ \text{dtw}(i-1,j-1) \end{array}\right\}+d(kp_{i},kp_{j}^{\prime })$$

(3) $$d_{\text{DTW}}(\text{Seq},{\text{Seq}}^{\prime })=\text{dtw}(t,u)$$

In order to obtain the weighted distance between key poses, $d(kp_{i},kp_{j}^{\prime })$, in a multi-view recognition, the previously obtained camera weights, *w_m,a_*, are employed. In this way, the relevance of each view can be employed to appropriately combine multi-view information. Since each action class has a different weight, we take the known class, *a*, of the training sequence, *Seq*, so as to choose the corresponding weight. In other words, we always suppose that the current comparison is the right match and therefore apply the appropriate weights, which indicate to what degree each view should be taken into account. The distance, $d(kp_{i},kp_{j}^{\prime })$, is obtained as follows:

(4) $$d(kp_{i},kp_{j}^{\prime })=\overset{M}{\underset{m=1}{\text{∑}}}w_{m,a}({\left\|{\bar{\text{V}}}_{m}-{\bar{\text{V}}}_{m}^{\prime }\right\|}_{1})$$

where *kp_i_* = ***V̄***_1_ ‖***V̄***_2_‖ … ‖ ***V̄****_M_* and $kp_{j}^{\prime }={\bar{\text{V}}}_{1}^{\prime }∥{\bar{\text{V}}}_{2}^{\prime }\left\|\ldots \right\|{{\bar{\text{V}}}^{\prime }}_{M}$.

**Figure 5.**
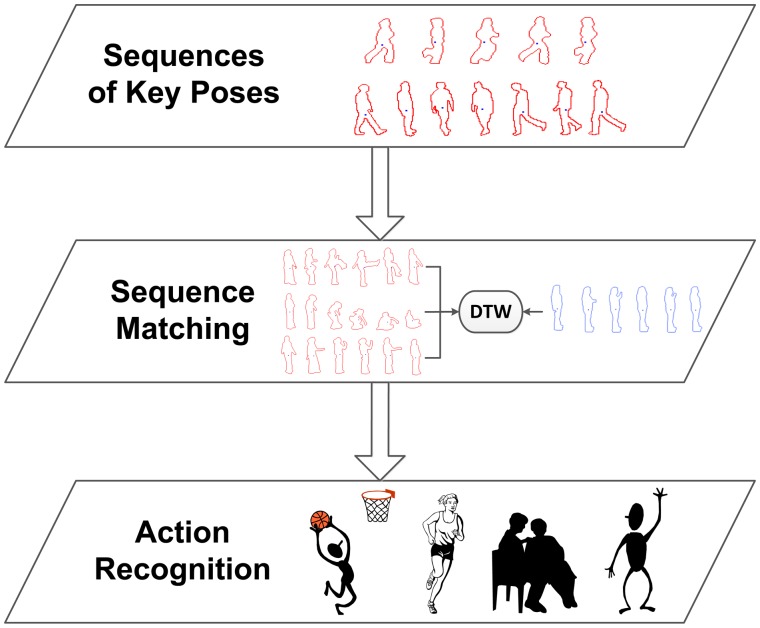
Outline of the recognition stage. The unknown sequence of key poses is obtained and compared to the known sequences. Through sequence matching, the action of the video sequence can be recognised.

Finally, recognition is performed by returning the label of the best match, *i.e.*, the instance with the lowest DTW distance. Figure 5 shows a diagram, where the recognition stages are detailed. As will be seen in Section 6, the presented contributions significantly improves the method's behaviour for multi-view scenarios, while, at the same time, a better temporal performance is obtained.

## 5. Privacy by Context

Before going into detail about the proposal for preserving privacy, we have to identify the elements that an individual may want to protect. An image conveys visual data that can be extracted and interpreted from a semantic level. This data provides visual information about identity, appearance, location, time and the ongoing activity. In Table 2, the visual clues related to this information can be seen. While this table is not intended to be exhaustive, through the presented visual clues, for instance, the identity of the person, or even the room where the person is, could be inferred. Furthermore, in the case of a sequence of images, additional information can be analysed, like gait, movement, gesture, action, activity, behaviour and the interaction with objects.

As can be seen, visual data exposes a lot of information about individuals appearing on images and videos. Individuals may want to conceal all of this data, but in this case, the remaining information would be useless for the AAL services that build upon it. For this reason, a privacy-by-context approach is proposed in order to fulfil the privacy protection requirement of the vision@home project. Specifically, a level-based visualisation scheme is presented. This approach is two fold. On the one hand, it aims to adapt privacy to the individual, because of the subjectivity involved in this matter. On the other hand, it enables one to balance information usefulness and the provided privacy in order to ensure that AAL services work properly. The adaptability of the privacy is provided by the context, whereas the balance is provided by visualisation levels that establish the way in which original images are modified and displayed.

By using this scheme, individuals are able to decide who, how and when they are watched. In other words, this proposal supports the privacy preferences of the involved individuals. Therefore, the context provides enough information in order to enable people to point out those situations in which privacy must be preserved. Regarding this, the following variables make up the context used in this proposal:

1. Identity of the subject (this is needed to retrieve the privacy profile)
2. Appearance (is the person dressed?)
3. Location (e.g., kitchen, bathroom, bedroom, *etc.*)
4. Ongoing activity (e.g., cooking, watching TV, sleeping, *etc.*)
5. The event
6. The observer and his/her access rights
7. Closeness between observer and subject (e.g., relative, doctor, friend, acquaintance, *etc.*)
8. Response of the subject (if it has been requested)

The detection of the context relies on the intelligent monitoring services for the recognition of all the variables, except Variables 6, 7 and 8, which are obtained with other means (e.g., directly retrieved from a database). The context is an essential part of the privacy scheme. Concerning this, if the context is not correctly recognised by the intelligent monitoring services, then privacy protection will fail. For instance, the corresponding visualisation level will not be established or it may be established in the wrong way. Furthermore, if the region of interest that contains the sensitive information is not recognised, privacy leaks will be produced, because the protection would be applied to a different region. Therefore, both the context and the sensitive regions must be properly detected in this approach.

Regarding visualisation levels, they only have a single constraint, *i.e.*, they must modify the raw image in such a way that privacy is preserved, and at the same time, the usefulness of the information is retained. The appropriate visualisation level will be dynamically selected according to the context. Furthermore, the association between an instance of the context and the visualisation level must be done beforehand by the individual in order to customise his/her privacy. Since the proposed privacy scheme is very generic, it leaves the definition of visualisation levels open. However, a set of visualisation levels could be defined depending on the application domain.

In this project, visualisation levels have been defined, taking into account the requirements of a remote observer and what an individual may want to keep private (see Table 2). A remote observer needs that certain information be available in the image in order to assess the gravity of a home accident, like a fall, and perform safety confirmation. The specific information includes posture, position (on the floor, on the bed, *etc.*), injuries, consciousness (voluntary movements, response to requests, *etc.*), vital signs (e.g., breathing), involuntary movements (tremors, muscle spasm, *etc.*) and facial expressions (e.g., to pain). Most of this information is related to only three visual clues: posture of the body, its appearance and movement. Through these clues, an observer could assess the gravity of a home accident. In this regard, facial expressions can also be useful in order to assess whether the individual has pain or not, whereas the position can help to evaluate the distance to objects that are involved in the accident.

**Figure 6.**
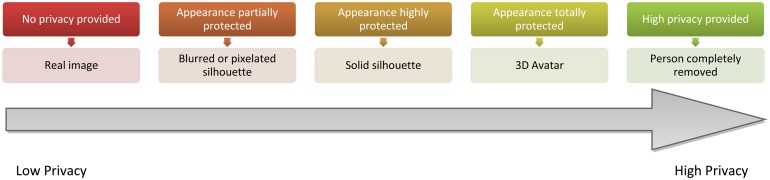
Specific visualisation levels that have been defined to apply the proposed privacy scheme to the vision@home project. The upper boxes indicate the protection level, whereas the lower boxes specify the proposed visualisation models.

Taking into account the aforementioned constraints, five visualisation levels have been defined for the vision@home project concerning the information that should be shown or protected. This proposal is focused on the foreground that corresponds to the persons in the image, which we consider the most sensitive region. The introduced visualisation levels modify the appearance of the individual, from low protected to highly protected. As Figure 6 details, the appearance of the person is protected in each level to a different degree, either with a blurring of the region corresponding to the person, a fully shaded silhouette or the replacement of the person with a 3D avatar, which imitates the same posture and movement as the person. Finally, the person can also be completely removed. This makes it possible to preserve the right to privacy of other inhabitants that are not the subject of the current monitoring for the AAL services of telerehabilitation and videoconferences. Regarding identity, while it is not protected in low protection levels (*i.e.*, the first two), it is in higher protection levels. Note that a silhouette may enable identification, due to height or other body features.

As can be seen, these levels are focused on protecting identity and appearance, leaving location and activity unprotected, since this data is necessary to provide the video-based telecare services. Nevertheless, location and activity are considered in the context to select the appropriate visualisation level.

**Figure 7.**
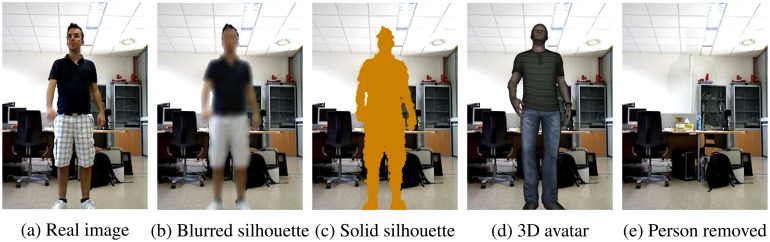
Visualisation models, ordered from lower to higher protection. These images have been obtained by applying the different models to the same video sequence.

As a result of the defined scheme, each visualisation level leads us to a model that provides the mentioned protection. An example of the developed visualisation models can be seen in Figure 7, where a different image redaction method is provided for each visualisation level.

## 6. Experimentation

Given that the proposed intelligent monitoring system relies mainly on the ability to understand human behaviour in order to provide proactive AAL services, in this section, the experimental results that have been obtained with the proposed human action recognition method are presented. These are compared with both the state-of-the-art and previous work.

Most state-of-the-art research works, as those mentioned in Section 2.2, include experimental results on publicly available datasets. This is useful and necessary in order to compare the different proposed approaches in terms of recognition accuracy. Nonetheless, most works only detail the results obtained on one or two specific datasets. We have found that further evaluations need to be performed in order to test the required robustness and generality, since commonly results vary significantly with respect to the type of data. In this sense, and as stated in [12], the suitability of current methods for human action recognition in real-world applications is still arguable. For this reason, so as to confirm the suitability of the present approach, in this paper, a wide experimentation is made, and performance results are included for all the tests.

In this section, our objective is to detail the performed experimentation and discuss the returned results. As mentioned before, tests have been performed on several human action datasets with different levels of difficulty, which is related to image quality, corresponding binary segmentations, the number of action classes and subjects, viewing angles, scenario-specific conditions, *etc.* Both single- and multi-view datasets have been employed. As input data, binary segmentations, obtained either manually or automatically (by means of background subtraction or depth sensors), have been tested.

The following types of cross-validation have been performed:

1. Leave-one-sequence-out (LOSO): In this cross-validation test, all the sequences, but one, are used for training, and the system is tested with the remaining one. This procedure is repeated for each sequence, and the final result is determined by the average accuracy score.
2. Leave-one-actor-out (LOAO): Similarly, in this test, the sequences of all, but one, actor are used for training, and the sequences of the unknown one are used for testing. Again, this is done for all the available actors, and the results are averaged. This test presents an additional difficulty, due to the inherent differences among subjects. Actor variance due to clothes, body build and the personal way in which each subject performs an action commonly leads to worse classification results.

Regarding the performed temporal evaluation, a test environment with the following characteristics has been employed:

1. Tests have been performed on a standard PC with an Intel Core 2 Duo CPU at 3 GHz and 4 GB of RAM running Windows 7 x64. The proposed method has been implemented using the .NET Framework and the OpenCV library.
2. Execution time has been measured using the hardware counter, QueryPerformanceCounter, with a precision of *μs*.
3. The temporal performance has been measured for the whole classification process, starting with the input of binary segmented images and going through contour extraction, feature extraction and key pose sequence matching.
4. Some optimisations have been implemented at the algorithmic level: (1) we have introduced thread-level parallelism to the *K*-means algorithm; and (2) we used an upper bound when searching for the nearest neighbour key poses.
5. The Manhattan distance has been chosen for feature comparison, since, in our experimentation, it improved the temporal performance over the Euclidean distance without compromising recognition accuracy.
6. No further optimisations or special purpose hardware have been employed.

Finally, the constant parameters of the algorithm (the number of radial bins, *B*, and key poses, *K*) have been chosen based on experimentation. The presented results have been obtained with *B* ∈ [10, 34] and *K* ∈ [4, 130]. Note that, in order to apply the proposed weighted feature fusion scheme from Section 4.1, the camera weights are learned using the same type of cross-validation test, but with single-view data.

### 6.1. Using RGB Data

In this subsection, the results that have been obtained on benchmarks consisting of traditional RGB video images are described.

#### 6.1.1. Weizmann Dataset

The Weizmann dataset [34] is among the most popular human action recognition datasets. It includes 93 video sequences of ten actions performed by nine actors, which have been recorded from a static front view. Binary segmentations obtained by means of background subtraction techniques are provided. Specifically, we employ the ones without post-alignment.

Most of the state-of-the-art approaches achieve perfect recognition on this dataset. Nonetheless, as we are targeting real-time applications, we included it as a baseline for single-view human action recognition and compared our performance with other fast approaches. Table 3 shows the obtained results. As is common in the state-of-the-art, we excluded the skip action and performed both LOAO and LOSO cross-validation tests. As can be seen, perfect recognition is obtained, and more importantly, the measured frame rate shows an outstanding suitability for real-time applications.

#### 6.1.2. MuHAVi Dataset

Since the proposed method targets multi-view human action recognition, the multicamera human action video dataset (MuHAVi) [40] has been included in the experimentation. It is one of the multi-view datasets with the highest image quality. The image resolution is 720 × 576 px, and manually annotated silhouettes are provided for a subset of two camera views (front-side and 45°). We tested both versions of the dataset with either 14 (MuHAVi-14) or the simplified eight (MuHAVi-8) action classes.

In order to provide a complete comparative analysis, we followed the authors guidelines and applied, in addition to the LOSO and LOAO cross-validations, a leave-one-view-out cross-validation (LOVO). Similarly to the other tests, with LOVO, view invariance is tested by using one view for training and the other for testing, and vice versa. Accordingly, single-view pose representations are employed in this test.

As Table 4 shows, in MuHAVi-8, perfect recognition has been obtained for the LOSO and LOAO cross-validation tests, and at the same time, a superior recognition speed is achieved. In the case of MuHAVi-14, the currently available recognition rates also have been significantly outperformed (see Table 5). Furthermore, even if our method is not intended for cross-view recognition, the behaviour of the proposed radial silhouette-based feature considering a 45° shift is promising. To the best of our knowledge, these are the highest results reported so far on this dataset.

#### 6.1.3. IXMAS Dataset

*The Institut National de Recherche en Informatique et en Automatique* Xmas motion acquisition sequences dataset (INRIA XMAS or IXMAS) [29] is the most popular multi-view human action recognition dataset. It includes the performances of 14 different actions performed by 12 actors, three times each. Five cameras have recorded the scenario from four side views and one top view. This leads to a dataset with over 2,000 video sequences. This benchmark presents an above-average difficulty, due to multiple reasons: (1) the important number of different actions and actors results in a higher inter-class similarity, which explains why the available methods commonly achieve lower recognition rates on this dataset than on the aforementioned ones; (2) the subjects chose their position and orientation towards the cameras freely, which implies that no view can be assigned as front or side and that this dataset is implicitly testing view invariance; and (3) a few actors performed actions differently, such as, for instance, with the opposite body part, which is why several authors decided to exclude some samples or actors.

Similarly to the case of the Weizmann dataset, we employ the automatically obtained binary segmentations, which are provided along with the video data. In contrast to the manually obtained silhouettes from the MuHAVi dataset, here, the resulting contours present a considerable amount of noise and incompleteness.

In our tests, we used the configuration that has been proposed by the authors of the dataset. Therefore, the point and throw actions have been excluded. Table 6 shows the comparison of our results with the state-of-the-art. The number of action classes, actors and views has been indicated, since test configurations vary. It can be observed that Yan et al. [43], Adeli-Mosabbeb *et al.* [22] and Cherla *et al.* [42] excluded the top view, and Wu *et al.* [44] obtained their best result excluding one of the side views. Recently, Holte *et al.* [45] achieved perfect recognition using a sophisticated method based on 4D spatio-temporal interest points and optical flow histograms. However, these recognition rates decrease when we look at approaches that target real-time applications. When comparing both the recognition rate and speed, our method stands out, achieving a 91.4% recognition accuracy at 207 FPS.

In order to give further insight about how the weighted feature fusion scheme considers the multiple views, in Table 7, the specific normalised weights of each view and action class are detailed. It can be observed that on average, Cam1 presents a slightly higher weight. This is related to the camera setup: Cam1 mostly recorded the front view. Its greater discriminative value leads to higher weights in general. Similarly, Cam4 recorded a top-view, which makes it difficult to distinguish some actions (such as sit down and get up). However, when taking a closer look at the action class-specific weights that have been assigned, considerable differences among the action classes show up. The fine-grained camera-action weights allow us to capture this condition and apply an appropriate feature fusion.

As has been mentioned before, the IXMAS dataset requires the classification algorithm to support the recognition from arbitrary views, *i.e.*, it cannot be assumed that a camera view only recorded a specific orientation of the subject. This is unavoidable in a real scenario, since subjects should be able to perform an action regardless of their orientation or location in the field of view. Regarding this matter, Cherla *et al.* [42] reorganised the available video images in six out of eight possible 45° orientations in order to achieve view consistency. So as to evaluate how much our result was influenced by the subject's orientations, we applied a similar configuration with seven side views and one top view. Interestingly, the obtained recognition rates were very close (±∼1%). This can be made clear looking closer at the learning stage of the algorithm. Even if a single camera view contains multiple orientations, the obtained key poses do represent these differences as long as samples are included in the training data. Therefore, inconsistent viewing angles can be recognised and, although our silhouette-based feature is not view-invariant, the learning algorithm itself handles this situation well and does not depend on fixed orientations.

### 6.2. Using Depth Data

Another important problem in silhouette-based human action recognition is data acquisition. The presented method relies on previously obtained human silhouettes and certainly depends on their quality and completeness. Commonly, human silhouettes are extracted based on background subtraction techniques that can perform in real time, although difficulties related to shadows and lighting remain [47]. Nevertheless, recent advances related to low-priced depth sensors, such as the Microsoft Kinect, allow one to obtain reliable depth information in real time. Therefore, we chose to validate the data acquisition stage of our proposal using the human silhouettes provided by these kinds of RGB-D sensors. Since we intend to provide AAL services at home, and the Microsoft Kinect shows itself to be proficient in indoor scenarios, this option suits us particularly well, both as a low-cost and real-time applicable solution. In this sense, the presented method has been tested on two datasets that contain depth data. The first one, the domotics and ambient intelligence group (DAI) RGBD dataset, has been recorded by ourselves in order to acquire multi-view depth data. In the case of the second one, a much larger publicly available dataset has been used.

Note that the presented visualisations models of the privacy-by-context method from Section 5 also rely on RGB-D information. By means of depth sensors, not only the human silhouette is extracted, but also a markerless 3D body pose estimation can be obtained. This pose estimation is employed in our proposal to animate the 3D avatar and place it in the same posture as the real person.

#### 6.2.1. DAI RGBD Dataset

**Figure 8.**
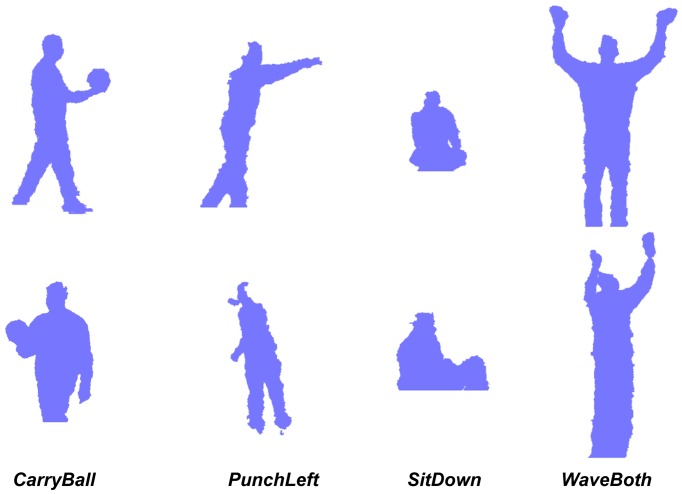
Sample silhouettes from our RGB-D dataset: front view at the top and backside view at the bottom.

In our setup, we used two Microsoft Kinect devices recording a front and a 135° backside view. Cameras were located at 2.5 and 3.5 m, respectively, recording an indoor scenario with halogen lighting. This dataset includes the following 12 actions classes: Bend, CarryBall, CheckWatch, Jump, PunchLeft, PunchRight, SitDown, StandingStill, Standup, WaveBoth, WaveLeft and WaveRight, where the StandingStill class can be interpreted as a null class. These actions have been performed by three male actors with noteworthy differences related to their body build. A few sample silhouettes are shown in Figure 8. These have been obtained using depth-based segmentation. In future works, we intend to publish an expanded version of this dataset with more types of human motion, like gestures, actions and activities of daily living (ADL), as well as more subjects and samples.

Table 8 shows the results that have been obtained using the depth-based silhouettes for both types of cross-validations. In order to provide further insight, the rates without the weighted feature fusion scheme, *i.e.*, using a simple feature concatenation considering all views equally, have been included. As can be seen, the *a priori* knowledge about the proficiency of each camera in recognising specific action classes leads to a substantial increase in recognition accuracy.

#### 6.2.2. DHA Dataset

The depth-included human action video dataset (DHA) [48] is aimed at providing a large dataset for human action recognition relying on single-view depth data, which is consistent with the traditional motion-based human action datasets, like Weizmann [34]. It contains 23 action classes performed by 21 different actors (12 males and nine female). Both colour and depth data are provided.

Specifically, we used the configuration that includes the ten actions from the Weizmann dataset and applied the LOSO cross-validation (see Table 9). The obtained recognition rate outperforms the baseline by 4.4%. Tests have also been performed on the complete dataset with all 23 action classes. In this case, our method reaches a success rate of 72.1%.

### 6.3. Conclusions

In conclusion, the performed experimentation shows that the proposed human action recognition approach reaches high recognition rates steadily. Furthermore, an outstanding proficiency for actor variance has been unveiled by the LOAO cross-validation tests. Regarding the temporal performance, a recognition speed that is significantly above the video frequency has been measured. Note that the varying recognition speeds are caused by the specific parametric configuration of the tests, as well as the different image resolutions. The main reasons for the advantage of our method regarding temporal performance are the employed feature representation and multi-view fusion. Both the single- and multi-view pose representations include a highly reduced number of elements, *i.e.*, respectively, *B* and *B* × *M* representative values. For this reason, our method is faster than other silhouette-based human action recognition methods with similar spatio-temporal matching. It is also worth mentioning that the contour extraction stage took on average ∼84% of the total processing time, which means that after the contour points have been obtained, the processing runs at over 1,000 FPS, resulting in an almost insignificant recognition time. This leads us to the conclusion that this method is suitable for the human behaviour analysis task of our intelligent monitoring system.

## 7. Discussion

In this contribution, the vision@home project has been presented focusing on its architectural design proposal, human behaviour analysis and privacy scheme. It can be observed that the main advances that have been made are related to the behaviour analysis based on action recognition. This essential part is required in order to both support the desired AAL services, as well as to enable the privacy-by-context method. In this sense, although a big step forward has been taken, still much work has to be done. Certainly, action recognition is just the first level of behaviour recognition that has to be targeted, and more complex activities can be recognised, as has been proposed in the design of this project. Since our proposal relies on a silhouette-based feature, only actions with considerable body motion can be detected. Nevertheless, gestures that are performed with the hands or the face also provide valuable clues about human behaviour. This type of motion could be modelled with the 3D information that is provided by depth cameras and allows body pose estimation. In fact, in previous work, we have already obtained successful results for gesture recognition relying on skeletal information and single-view setups [49]. Furthermore, as has been mentioned beforehand, the context depends, as well, on scene understanding. This implies not only behaviour recognition from one and multiple subjects, but also recognition of objects and interactions, as well as person identification and the recognition of nudity. Based on the values of these context variables, the privacy scheme has to be able to determine the appropriate visualisation level that is suitable for that particular scenario and circumstance. For this purpose, a generic association could be proposed based on the default instructions of healthcare professionals and people's statistical preferences. An adaptive learning method can then be employed to customise the selection of visualisation levels based on the inhabitant's interaction history. To consider and combine this kind of information requires a significant effort, which has to be addressed in future work.

Regarding the technical constraints of the proposals that have been made, the experimental results have shown that a considerable generality has been reached in order to support different test scenarios, subjects and actions, as well as visual sensors. However, the validation has to be performed on real-world scenarios, especially since privacy has been shown to be subjective to the individual. In this sense, experts and healthcare professionals have to be consulted in order to perform *in situ* validation and to interpret the received feedback appropriately.

In this sense, even if huge steps have to be made to reach the final deployment of these kinds of intelligent monitoring systems at home, the vision@home project proposes a feasible solution to enable vision-based human behaviour analysis and telecare at home. This, in turn, leads to the support of AAL services, which are of great value for the inhabitants.

## 8. Conclusions

In this paper, a vision-based intelligent monitoring system designed to support AAL services has been presented. As part of the vision@home project, the desired services, requirements and resulting design proposal have been specified. This has led to a system that relies mainly on human behaviour analysis in order to offer care and safety services to support personal autonomy. In this sense, behaviour recognition based on human action recognition is considered, where, based on previous work, novel contributions have been made to support multi-view scenarios. Our method provides efficient handling of multi-view scenarios by means of feature fusion. Different camera setups for training and testing stages are supported, and no previous calibration is required. Furthermore, the usefulness of the different viewing angles is explicitly taken into consideration based on a weighted fusion approach. Experimental results show that this approach achieves accurate recognition and the required real-time performance through a variety of publicly available datasets, where existing fast approaches are significantly outperformed and new reference recognition rates are established. In order to validate the data acquisition stage of the method, which has to provide the human silhouette, depth sensors have been tested, obtaining outstanding results. Moreover, in order to provide telecare services, such as telerehabilitation and safety confirmation, visual privacy protection techniques are considered. Particularly, a privacy-by-context method has been introduced. For this purpose, the available visual clues have been analysed and the context variables have been established, taking also into account the information that has to be retained to provide the AAL services. Specifically, a privacy scheme has been presented that focuses on the image of the person and establishes different visualisations levels that protect the appearance of the person according to the context. For this project, five particular visualisation levels and associated models have been proposed in order to protect identity and appearance based on image redaction methods.

Therefore, in this project, a feasible solution is given to provide AAL services and to support the independent living at home of elderly and impaired people by means of visual monitoring techniques, while preserving the right to privacy of the inhabitants.

This work has opened multiple future directions, which can be explored to improve its performance, extend its applicability to other applications or scenarios or further study its evaluation and final deployment. In general, however, two main hurdles that have to be overcome can be distinguished. On the one hand, as has been stated before, based on sequences of actions and other multi-modal data from environmental sensors, more complex activities can be recognised. This would allow one to increase the degree of semantics and the time frame of the analysis, leading to more advanced behaviour analysis and enabling valuable health-status monitoring services. On the other hand, an early validation of the current advances in real scenarios should be carried out in order to adapt ongoing research and new proposals to the deficiencies that may be observed.

## Figures and Tables

**Table 1.** A comparison of single-view recognition rates obtained on the IXMAS dataset. The result of the Leave-one-actor-out (LOAO) cross-validation is shown (bold indicates the highest). Note that Cam1 corresponds to the front view in most samples.

**Cam0**	**Cam1**	**Cam2**	**Cam3**	**Cam4**
67.7%	**78.8%**	69.9%	75.0%	55.1%

**Table 2.** The types of information that can be extracted out of image sequences and the related visual clues that can provide this information.

**Information**	**Related Visual Clues**
Who is the person? (identity)	Face
	Hair
	Skin
	Height
	Clothes
	Gait

How is the person displayed? (e.g., the appearance)	Face expressions
	Hair (e.g., hairstyle, colour, *etc.*)
	Body (e.g., nudity)
	Posture
	Shape
	Colour

Where is the person? (location)	Room
	Spatial position (e.g., on the floor, on the bed, *etc.*)
	Room signs

What is the person doing? (ongoing activity)	Behaviour (*i.e.*, movement, gesture, action, activity)
	Gaze
	Spatial position
	Objects and interactions

When is the activity taking place? (time)	Temporal clues (e.g., a wall clock)

**Table 3.** Comparison of recognition rates and speeds (in frames per second (FPS)) obtained on the Weizmann dataset with other state-of-the-art approaches. LOSO, leave-one-sequence-out.

**Approach**	**Test**	**Rate**	**FPS**
İkizler and Duygulu [35]	LOSO	100%	N/A
Tran and Sorokin [24]	LOSO/LOAO	100%	N/A
Fathi and Mori [36]	LOSO	100%	N/A

Cheema *et al.* [37]	LOSO	91.6%	56
Chaaraoui *et al.* [32]	LOAO	92.8%	124
Sadek *et al.* [38]	LOAO	97.8%	18
Guo *et al.* [39]	LOSO	97.8%	40

Our approach	LOSO/LOAO	100%	197

**Table 4.** Comparison of recognition rates and speeds obtained on the MuHAVi-8 dataset with other state-of-the-art approaches. LOVO, leave-one-view-out.

**Approach**	**LOSO**	**LOAO**	**LOVO**	**FPS**
Singh *et al.* [40]	97.8%	76.4%	50.0%	N/A
Eweiwi *et al.* [41]	98.5%	85.3%	38.2%	N/A

Cheema *et al.* [37]	95.6%	83.1%	57.4%	56
Chaaraoui *et al.* [31]	98.5%	95.6%	-	66

Our approach	**100%**	**100%**	**82.4%**	**98**

**Table 5.** Comparison of recognition rates and speeds obtained on the MuHAVi-14 dataset with other state-of-the-art approaches.

**Approach**	**LOSO**	**LOAO**	**LOVO**	**FPS**
Singh *et al.* [40]	82.4%	61.8%	42.6%	N/A
Eweiwi *et al.* [41]	91.9%	77.9%	55.8%	N/A

Cheema *et al.* [37]	86.0%	73.5%	50.0%	56
Chaaraoui *et al.* [31]	94.1%	86.8%	-	51

Our approach	**98.5%**	**94.1%**	**59.6%**	**99**

**Table 6.** Comparison with other multi-view human action recognition approaches of the state-of-the-art. The rates obtained in the LOAO cross-validation performed on the IXMAS dataset are shown (except for [42], where the type of test is not stated).

**Approach**	**Actions**	**Actors**	**Views**	**Rate**	**FPS**
Yan *et al.* [43]	11	12	4	78%	N/A
Adeli-Mosabbeb *et al.* [22]	10	11	4	87.5%	N/A
Wu *et al.* [44]	12	12	4	89.4%	N/A
Cilla *et al.* [23]	11	12	5	91.3%	N/A
Weinland *et al.* [29]	11	10	5	93.3%	N/A
Cilla *et al.* [26]	11	10	5	94.0%	N/A
Holte *et al.* [45]	13	12	5	100%	N/A

Cherla *et al.* [42]	13	N/A	4	80.1%	20
Weinland *et al.* [46]	11	10	5	83.5%	∼500
Chaaraoui *et al.* [32]	11	12	5	85.9%	26

Our approach	11	12	5	91.4%	207

**Table 7.** Camera weights that have been obtained for the five views of the IXMAS dataset using the proposed weighted feature fusion scheme.

**Action**	**Cam0**	**Cam1**	**Cam2**	**Cam3**	**Cam4**
*check watch*	0.18	0.24	0.19	0.18	0.21
*cross arms*	0.16	0.23	0.21	0.20	0.20
*scratch head*	0.17	0.21	0.20	0.23	0.19
*sit down*	0.23	0.23	0.21	0.23	0.10
*get up*	0.22	0.23	0.21	0.23	0.11
*turn around*	0.20	0.21	0.20	0.21	0.18
*walk*	0.22	0.21	0.20	0.21	0.16
*wave*	0.20	0.25	0.16	0.16	0.23
*punch*	0.20	0.20	0.23	0.18	0.19
*kick*	0.21	0.26	0.17	0.25	0.11
*pick up*	0.21	0.21	0.21	0.22	0.15

**Average**	0.20	0.23	0.20	0.21	0.17

**Table 8.** Cross-validation results obtained on our multi-view depth dataset.

**Approach**	**LOSO**	**LOAO**	**FPS**
Feature concatenation	83.3%	94.4%	80
Weighted feature fusion scheme	**94.4%**	**100%**	80

**Table 9.** LOSO cross-validation results obtained on the depth-included human action (DHA) dataset (10 Weizmann actions).

**Approach**	**LOSO**	**FPS**
Lin *et al.* [48]	90.8%	N/A *^a^*
Our approach	**95.2%**	99

a An average descriptor extraction time of 3.9 s per video sequence is stated.
